# A simple modification to the 25-gauge trocar and cannula system for retinopathy of prematurity related lens-sparing vitrectomy

**DOI:** 10.1186/s12886-016-0214-4

**Published:** 2016-04-12

**Authors:** Ian Y. Wong, Lawrence P. Iu, Connie H. Lai

**Affiliations:** Department of Ophthalmology, LKS Faculty of Medicine, The University of Hong Kong, Room 301, Level 3, Block B, Cyberport 4, Pokfulam, Hong Kong

## Abstract

**Background:**

Recently, 25-gauge vitrectomy has become more popular. However, most still perform the surgery in pediatric patients without the use of the trocars and cannulas as in adult vitrectomies.

**Methods:**

We described a simple modification using adult 25-gauge cannulas and 270-silicone watzke sleeves, enabling these instruments to be used in pediatric cases. The sleeve is cut into segments of 2 mm in length, and then introduced up the shaft of the 25-gauge trocar. One is introduced first, and a second one is introduced on top of the first one. This secures the two sleeves on the shaft of the trocar, such that they act as a spacer. The effective shaft of the trocar was then reduced to 2 mm in length.

**Results:**

This method enabled successful surgery in two cases.

**Conclusions:**

This allows the adaptation of the standard 25-gauge system for pediatric cases with only the slightest modification needed.

**Electronic supplementary material:**

The online version of this article (doi:10.1186/s12886-016-0214-4) contains supplementary material, which is available to authorized users.

## Background

One of the most undesirable complication in phakic vitrectomy is the inadvertent lens touch from instruments such as the trocar and cannular in 23-gauge or 25-gauge vitrectomy systems [1]. In adult cases, where the anatomy of the eye is more developed, such risks are lower. However, in pediatric cases, where the anatomy is not yet well developed, the chances of inadvertent lens touch cannot be underestimated [2]. The reasons behind this is due to a combination of 1) lens occupying a larger proportion in the posterior segment as compared to that in an adult; 2) the eye is much shorter than that of an adult. It can be as short as 16.5 mm in a newborn; 3) the under-development of the pars plana, where safe sclerotomies should be placed at 1 to 1.5 mm behind the limbus, rather than at 4 mm behind the limbus in an adult. These measurements are often so small compared to the size of regular instruments. For instance, the diameter of a 20-gauge cannula is 0.91 mm; 23-gauge is 0.64 mm; and 25-gauge is 0.51 mm. Moreover, because of the proximity of the lens and the sclerostomy site, the regular 4 mm-lengthed cannulas may risk lens touch if used as is.

We have invented a modification to the standard 25-gauge trocar and cannula system for adults, such that these could be used in pediatric cases, especially those with retinopathy of prematurity related lens-sparing vitrectomies.

## Methods

**Fig. 1:**
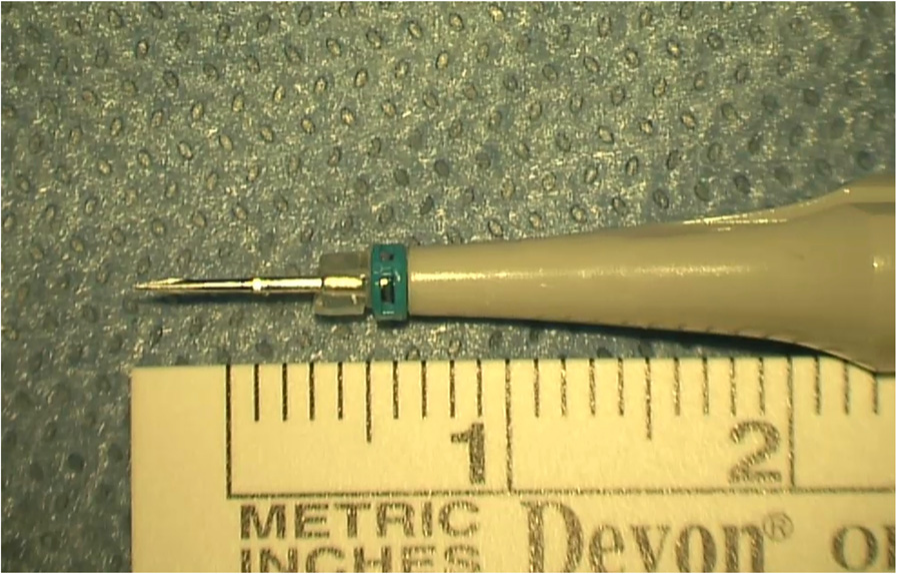
The resultant shaft of cannula has only 2 mm left which can be introduced into the eye

We used standard 25-gauge trocars and cannulas (25+ system, Alcon labs, Forte Worth, Texas, USA), and standard 270-silicone Watzke sleeves (Labtician, Oakville, Ontario, Canada). The 270 sleeves are cut using regular scissors, into spacers of 2 mm length (Fig. 1). One spacer will first be introduced up the cannula, such that only 2 mm of the cannula will be exposed, while the proximal 2 mm of it will be covered by the silicone spacer. The entire procedure is demonstrated in the Additional file 1: Video.

Next, a second spacer will be introduced onto a sleeve spreader, and introduced over the first one. The spreader will then be retrieved, leaving behind the second spacer overlaid on top of the first one. This converts the originally 4 mm-length cannula into a 2 mm cannula, with the spacers firmly attached to the cannula.

The trocars and cannulas are then inserted through the sclera at 1 mm behind the limbus, as in regular adult vitrectomies. There were 2 minor modifications during the insertion, 1) the angle of insertion was parallel to the visual axis to avoid lens touch, and 2) the trocars were inserted perpendicular to the sclera, and sutures were placed after removal at the end of surgery. The rest of the vitrectomy is the same as would be in an adult. The detailed procedure is shown in the video file. 

## Results

This technique was used in two cases where lens-sparing vitrectomies were performed for retinopathy of prematurity related retinal detachments. We employed this technique in all three ports during the surgeries, and it went well without any lens touch. In one case, one of the ports came out during vitrectomy with the cutter, but did not came out again after being re-fitted into place. This could be avoided by removing the cutter slowly as suggested by Babu et al. [3].

## Discussion

At 1 mm behind the limbus, the thickness of the sclera is roughly 0.5 mm [4]. Theoretically, any cannulas longer than that would be able enter the vitreous cavity. The current method reduced the length of the cannulas to only 2 mm, allowing entry into the vitreous cavity with a sturdy anchor on the sclera, and at the same time not too long to risk inadvertent lens touch. Common infusion lines available are usually of the length 1.5 mm or 2.5 mm long. This technique also allows the infusion to be connected in the same way as with the working ports.

Some surgeons would perform 25-gauge vitrectomies on similar cases without the use of the cannulas. This has the disadvantages in that the conjunctiva has to be opened, and the instruments have to go in through the bare sclerostomy, which can be difficult in such a small eye. This technique allows easy entry of the instruments and enjoyment of the advantages of the trocar and cannula system has to offer. In addition, the conjunctiva does not need to be dissected.

Babu et al have reported another method of using a trimmed 42-silicone band to act as spacer, and using it to shorted the length of the 25-gauge cannulas used [3]. During the piercing of the 42-silicone bands, we find that the trocar would create a crack in the band. During the maneuvers intra-operatively, this crack may theoretically extend and lead to dislodging of the spacer, with resultant inadvertent advancement of the cannula into the globe.

With the current technique, there are not cracks in the silicone spacer, cut out from a 270-silicone band. The inner luminal diameter of a 270-silicone band is 0.76 mm, which the outer diameter of a 25-gauge cannula is 0.51 mm. Although the spacer could not provide a snug fit onto the cannula, the second overlaying spacer would squeeze onto the first one, creating a stable fit on the cannula without chance falling off spontaneously. As 23-gauge cannulas are 0.64 mm in diameter, which is smaller than the inner luminal diameter of the spacer, this technique may also be adopted on the 23-gauge system.

Another advantage of this method is that all the involved items are readily available, and the 270-silicone sleeve is cheap in cost. This allows the adaptation of the standard 25-gauge system for pediatric cases with only the slightest modification needed. This would be of most benefits for centers that are not equipped with vitrectomy instruments tailor-made for pediatric vitrectomies. One disadvantage of this method over the one described by Babu et al [3] is that the current method is more time consuming. However, preparation of the instrumentations can be performed prior to the initiation of the surgery to avoid unnecessary idling of the patient.

## Conclusion

The novel method allowed conventional adult-sized vitrectomy instruments to be converted for use in pediatric cases with minimal maneuvers. This is useful for centers where pediatric case load is not high enough to justify purchasing a separate set of pediatric vitrectomy instruments.

### Ethical approval and consent to participate

Ethical approval has been obtained by the Institutional Review Board of the University of Hong Kong/Hong Kong West Clusters of Hospitals. Consent has been obtained from the parents of the patient for publication purposes (patient is a premature infant).

### Availability of data and materials

Data will not be shared as this involves privacy of the involved patient(s).

## Additional file

Additional file 1: Video showing how the modification is performed. Movie clip showing how the procedure is performed. First, the sleeve is cut into 2 mm long segments. This will act as the spacer. With the help of a spreader, 2 spacers are introduced onto the shaft of the cannula, one on top of the other. In the end the shaft of the 25-gauge cannula is reduced by 2 mm. (M4V 13084 kb)
